# Supplementary material for: Rapid Detection of Cd2+ Ions in the Aqueous Medium Using a Highly Sensitive and Selective Turn-On Fluorescent Chemosensor
Source: Molecules. 2023 Apr 21;28(8):3635. doi: 10.3390/molecules28083635 (PMC10143290; doi:10.3390/molecules28083635)
Supplement: Supplementary file 1 [file molecules-28-03635-s001.zip › molecules-2270372-supplementary.pdf]

Supplementary data

Rapid detection of Cd^{2+} ions in the aqueous medium using a highly sensitive and selective turn-on fluorescent chemosensor

Maria Sadia[1], Jehangir Khan[1], Rizwan Khan[2], Abdul Waheed Kamran[1], Muhammad Zahoor*[, 3], Riaz Ullah[4], Ahmed Bari[5] and Essam A. Ali[6]

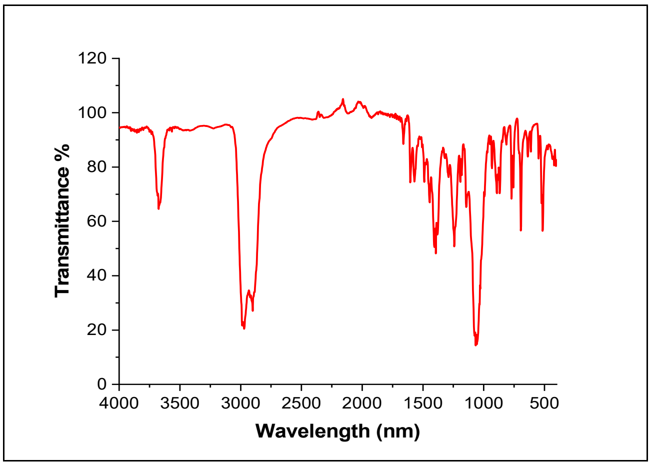

Figure S1: FT-IR spectrum of chemosensor **CM1**

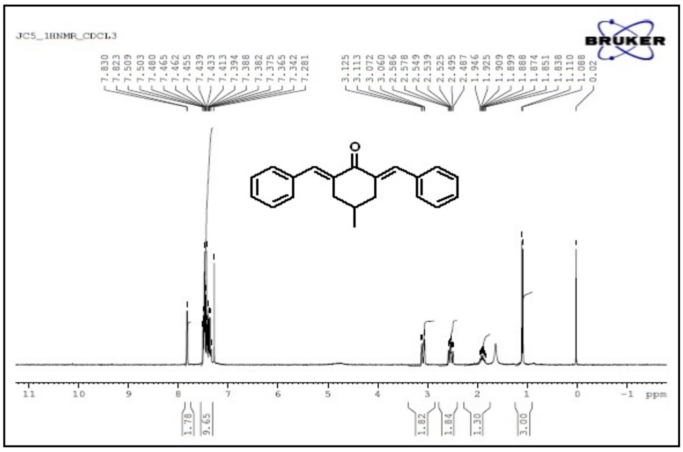

Figure S2: ^{1}H-NMR spectrum of the chemosensor **CM1**

Binding stoichiometry

The CM1-Cd^{2+} complex stoichiometry was investigated through Job's plot analysis based on fluorescence emission of Cd^{2+} and CM1 volume ratio, keeping the final volume constant. The maximum fluorescence emission intensity was observed when 0.6 molar fractions of CM1

reached, indicating 2:1 binding stoichiometry between chemosensor CM1 and Cd^{2+}. As shown in (**Figure S3**).

In Job’s plot analysis, the exact concentration of the reagents is unknown but if the concentration of one of the reagents cannot be measured precisely, the Job's plot can still be used as a graphical method to determine the stoichiometry of the metal-ligand complex [60, 61]. However, the accuracy of the stability constant determination using the Job's plot may be limited due to the binding stoichiometry and the effects of competing reactions or solution conditions that may affect the absorbance measurements.

Therefore, accurate measurement of the concentration of all reagents involved in the reaction is crucial to obtain a precise determination of the stability constant, and the Job's plot should only be used as a complementary method when the exact concentrations of the reagents are unknown or difficult to measure accurately.

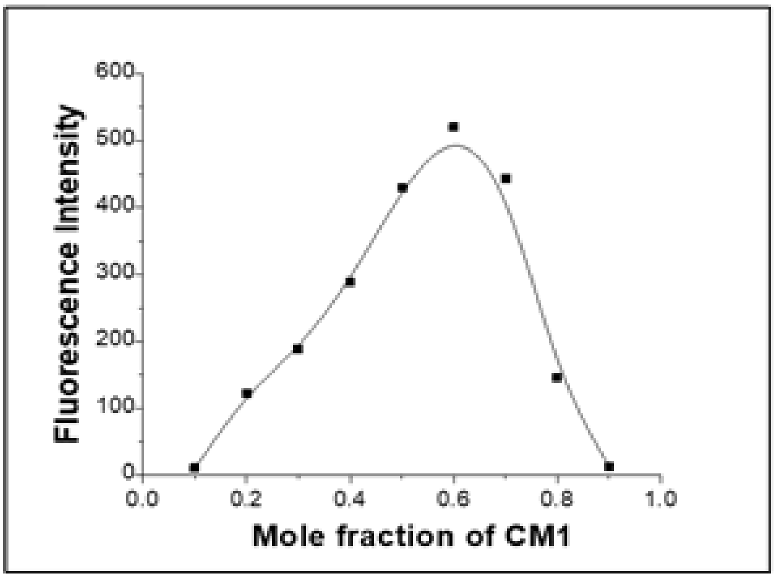

Figure S3: Job’s plot analysis of CM1 shows 2:1 binding ratio between chemosensor CM1 and Cd^{2+}

References

60. Mehta, P. K., Hwang, G. W., Park, J., & Lee, K. H. (2018). Highly sensitive ratiometric fluorescent detection of indium (III) using fluorescent probe based on phosphoserine as a receptor. *Analytical chemistry*, *90*(19), 11256-11264.
61. Wu, X., Niu, Q., & Li, T. (2016). A novel urea-based “turn-on” fluorescent sensor for detection of Fe^{3+}/F^{-} ions with high selectivity and sensitivity. *Sensors and Actuators B: Chemical*, *222*, 714-720.
